# Transcribed B lymphocyte genes and multiple sclerosis risk genes are underrepresented in Epstein–Barr Virus hypomethylated regions

**DOI:** 10.1038/s41435-019-0089-5

**Published:** 2019-10-16

**Authors:** Lawrence T. C. Ong, Grant P. Parnell, Ali Afrasiabi, Graeme J. Stewart, Sanjay Swaminathan, David R. Booth

**Affiliations:** 1 Centre for Immunology and Allergy Research, Westmead Institute for Medical Research, The University of Sydney, 176 Hawkesbury Rd, Westmead, NSW 2145 Australia; 2 Department of Clinical Immunology and Allergy, Westmead Hospital, Cnr Darcy and Hawkesbury Rds, Westmead, NSW 2145 Australia

**Keywords:** DNA methylation, B cells

## Abstract

Epstein–Barr Virus (EBV) infection appears to be necessary for the development of Multiple Sclerosis (MS), although the specific mechanisms are unknown. More than 200 single-nucleotide polymorphisms (SNPs) are known to be associated with the risk of developing MS. About a quarter of these are also highly associated with proximal gene expression in B cells infected with EBV (lymphoblastoid cell lines—LCLs). The DNA of LCLs is hypomethylated compared with both uninfected and activated B cells. Since methylation can affect gene expression, and so cell differentiation and immune evasion, we hypothesised that EBV-driven hypomethylation may affect the interaction between EBV infection and MS. We interrogated an existing dataset comprising three individuals with whole-genome bisulfite sequencing data from EBV transformed B cells and CD40L-activated B cells. DNA methylation surrounding MS risk SNPs associated with gene expression in LCLs (LCLeQTL) was less likely to be hypomethylated than randomly selected chromosomal regions. Differential methylation was independent of genomic features such as promoter regions, but genes preferentially expressed in EBV-infected B cells, including the LCLeQTL genes, were underrepresented in the hypomethylated regions. Our data does not indicate MS genetic risk is affected by EBV hypomethylation.

## Introduction

Epstein–Barr Virus (EBV) is a gamma herpesvirus that has been implicated as an etiological factor in several autoimmune and malignant conditions. Although most people have life-long infection with this virus, infection is usually under sufficient immunological control that there are no pathogenic consequences. In immunocompromised individuals, or those with certain mutations, EBV infection and proliferation can cause significant immune perturbation and death [1]. Suboptimal control of EBV infection may underpin the development of autoimmune disease and malignant conditions.

Multiple sclerosis (MS) is an autoimmune and neurodegenerative condition that affects the central nervous system, causing demyelination and progressive disability. Although most healthy individuals will become seropositive for EBV (80–90%), almost all MS-affected individuals are seropositive [2]. This suggests that EBV infection is necessary, but not sufficient for the development of MS and that interactions with other risk mechanisms might be necessary for disease. At present, over 200 single-nucleotide polymorphisms (SNPs) have been found to be associated with increased susceptibility to MS [3], and the vast proportion of the genes proximal to these SNPs are associated with immune cell pathways, and are associated with altered gene expression in immune cells of the blood [4]. Many of the MS risk SNPs have a stronger or different association with gene expression in EBV-infected B cells than blood [5]. The altered expression of risk genes appears in turn to affect infected B cell functions, including cell proliferation, and immune system evasion. Genetic variation due to somatic variation in genes affecting these functions has also been identified in nasopharyngeal carcinomas caused by EBV [6]. The MS risk SNPs may affect gene expression through altering transcription factor control of gene expression, through direct mechanisms such as affecting binding motifs, or through indirect mechanisms, through interaction with other gene regulatory processes.

One of the potential indirect mechanisms is through the effects of EBV infection on host DNA methylation. DNA methylation is a relatively stable epigenetic modification that is associated with changes in gene transcription, particularly at transcription start sites and promoter regions. An interaction between risk polymorphisms and EBV methylation may lead to abnormal activation, proliferation or persistence, and so disease.

EBV infection is known to cause widespread changes in genomic DNA methylation. Genome-wide studies of lymphoblastoid cell lines (LCLs; EBV transformed B lymphocyte lines) have mostly found DNA methylation to be decreased relative to comparator subsets such as whole blood cells [7, 8] and peripheral blood lymphocytes or leukocytes [9] perhaps due to decreased DNMT1 expression [10]. Studies comparing the DNA methylation of B cells and LCLs are more robust at detecting changes due specifically to EBV infection, as they are not confounded by cell lineage-specific methylation differences. Although these studies are limited [10–12], they have found LCLs demonstrate hypomethylation of promoter regions corresponding to B cell biological pathways compared with resting B cells [12]. In addition, EBV infection tends to increase DNA methylation in high CpG content promoters and decrease DNA methylation in low CpG content promoters [10]. Finally, EBV infection has been found to result in hypomethylation of over two-thirds of the entire genome [11].

We therefore hypothesised that EBV-driven hypomethylation may affect the interaction between EBV infection and MS. Specifically, we predicted that if hypomethylation contributes to the dysregulation of infected B cells leading to MS, MS risk loci would be overrepresented in these hypomethylated regions, especially the subset of these risk loci known to correlate with gene expression in LCLs.

## Materials and methods

### Datasets, alignment, and methylation calling

We utilized extant whole-genome bisulfite sequencing data from LCLs, CD40L-activated B cells, and resting B cells from three individuals [11]. Resting B cells have very different transcriptomes compared with LCLs and activated B cells. Therefore, unless otherwise specified, methylation of CD40L-activated B cells was compared with LCLs to distinguish between the effects of proliferation due to virus and B cell activation. The quality of raw sequences was ascertained using FastQC v0.11.7 [13]. Adapter trimming was then carried out using Trimmomatic v0.36 [14] with HEADCROP (set to 3) and MAXINFO (target length = 40, strictness = 0.5) options. Reads were aligned to the hg19 genome using the Wildcard Alignment Tool (WALT) v1.0. Modules from Methpipe v3.4.3 [15] were then used to process aligned files. Firstly, .sam files from the alignment were converted to .mr format using the *to-mr* module. Duplicate removal was performed using *duplicate-remover*, followed by estimation of bisulfite conversion rates using *bsrate*. Methylation calls were performed using *methcounts* with the -n option, to limit calls to CpG context cytosines only. *Methcounts* data were then merged using *merge-methcounts* for biological replicates within each condition i.e., LCL, CD40L-activated B cells, and resting B cells. Coverage data was determined using the *levels* module. Because CpG methylation is most commonly symmetric, the *symmetric-cpg* module was then used to merge methylation data from both strands prior to further analysis. Regional methylation analysis was performed using the *roimethstat* module (with -P and -v options), which determines the average methylation state within a prespecified region of interest.

### Regions of interest

We performed tiling analysis using 1-kb tiles centred upon MS risk loci identified previously by the International MS Genetics Consortium as being associated with increased disease risk [16] (MSGWAS). We also chose a subset of these risk loci which have been previously found to be associated with gene expression in LCLs [5] (LCLeQTL). We compared the number of differentially methylated regions (DMRs) with regions centred upon an unbiased list of single nucleotide polymorphisms from the NHGRI-EBI Catalog of published genome-wide association studies [17] (GWAS Catalog; downloaded 8 September 2018). Whole-genome 1-kb tiles were derived using Bedtools (v2.25.0) *makewindows* with *-w* set to 1000, based on hg19 coordinates. Significant variant-gene association lists were downloaded from https://storage.googleapis.com/gtex_analysis_v7/single_tissue_eqtl_data/GTEx_Analysis_v7_eQTL.tar.gz and the loci with the most significant associations (nominal *p* value < 10^−20^) extracted for whole blood (Whole_Blood.v7.signif_variant_gene_pairs.txt.gz) and LCLs (Cells_EBV-transformed_lymphocytes.v7.signif_variant_gene_pairs.txt.gz). Promoter regions were defined by the Ensembl regulatory build [18] and downloaded from ftp://ftp.ensembl.org/pub/grch37/current/regulation/homo_sapiens/. Exon and intron coordinates were derived from UCSC Genome Browser Table Browser > group: Genes and Gene Predictions > track: UCSC Genes > table: knownGene. Regions of the genome not covered by promoters, introns, or exons were considered to be intergenic.

### Differential methylation

DMRs were determined by taking subset-specific methylation values for a region of interest (as determined by *roimethstat*) and subtracting them from each other. An absolute methylation difference of >0.2 was used as the threshold for calling a DMR. Bedtools v2.25.0 was used for analysis of genomic features [19] and statistical analyses were performed using R statistical software and GraphPad Prism 8.0.0. Sex, mitochondrial, and haploid chromosomes were excluded from analyses.

### Proximal genes

Genes proximal to an MS risk locus, the midpoint of a 1-kb tile or a region of interest were determined by using the *closest* module of Bedtools v2.25.0.

### RNA sequencing and analysis

To correlate EBV-induced DNA methylation alterations with gene expression, we performed global gene expression profiling by RNA-seq on paired resting B cells and LCL samples from five individuals as previously described [5]. The raw and processed sequencing data generated are available from the NCBI Gene Expression Omnibus under accession number GSE126379. Log2 of normalized relative gene expression levels (logFC) was correlated with the methylation difference between LCL and resting B cells at exons, introns, and promoters proximal to these genes. Where more than one gene was proximal to an annotation, one gene was selected at random as the proximal gene.

## Results

Bisulfite conversion rates and coverage data following sequence alignment and duplicate removal can be found in Supplementary Information 1 and 2. In summary, the mean bisulfite conversion rate was 98.3% for LCLs, 98.4% for activated B cells, and 96.9% for resting B cells. Mean coverage was 5.7× for LCLs, 5.8× for activated B cells, and 5.5× for resting B cells. We used a methylation difference of >0.2 as the threshold for identifying DMRs.

### LCLs demonstrate widespread hypomethylation relative to activated B cells

**Fig. 1:**
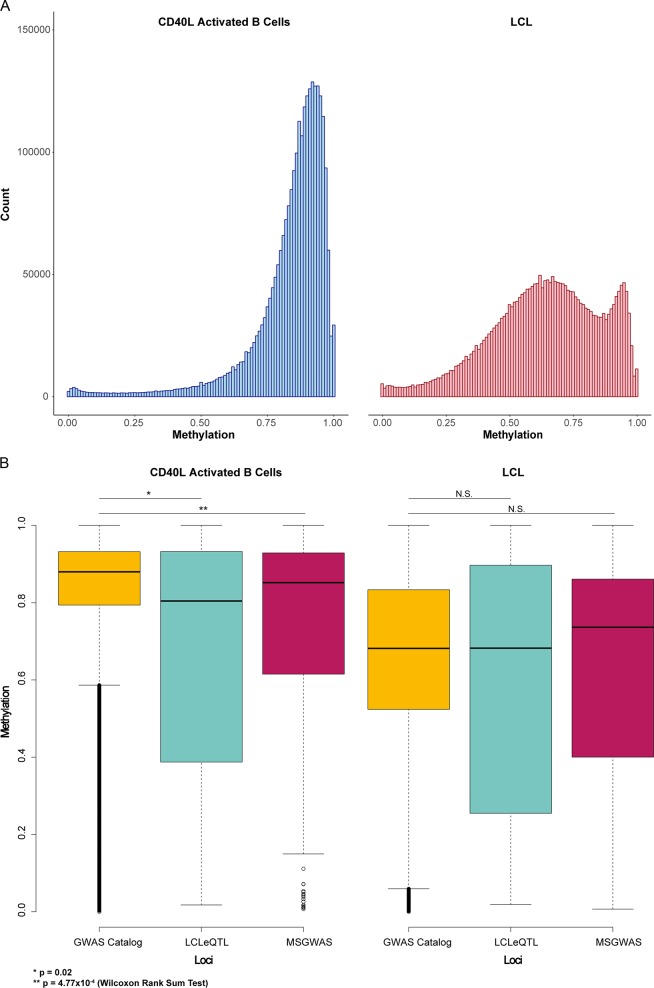
**a** distribution of methylation values of 1-kb tiles across the CD40L-activated B cell and LCL methylome. **b** Boxplot of DNA methylation by loci list and subset. Only CD40L-activated B cell tiles demonstrated significant differences when compared with regions centred on GWAS Catalog SNPS, suggesting that LCLeQTL and MSGWAS loci are less methylated even prior to EBV transformation

To confirm widespread hypomethylation found previously by Hansen et al. [11], we performed tiling analysis using 1-kb tiles across the genome as DNA methylation has previously been found to correlate across this scale [20, 21]. We found 45.9% of tiles to be hypomethylated in LCLs and 0.07% to be hypermethylated in LCLs. This was consistent with a left-shift in the distribution of methylation values in LCLs (see Fig. 1a).

### Differentially methylated regions are underrepresented at immune and B cell loci

There was a profound underrepresentation of DMRs when comparing MSGWAS and LCLeQTL loci with tiles generated from GWAS Catalog SNPs and genome wide 1-kb tiles (Table 1). To determine whether the observed number of DMRs were significantly different from chance, we randomly sampled 34 and 199 tiles from the GWAS Catalog loci 100 times and found the minimum number of DMRs to be 7 (20.59%) and 63 (31.66%), respectively, suggesting the results obtained would occur less than 1 in 100 times by chance alone. Hansen et al. [11] indicated previously that genes within hypomethylated blocks tended to be hypervariable with regard to expression. Using the same normalized LCL expression data [22], we found nine genes associated with differentially methylated LCLeQTL loci, three of which had expression level data. Of these, only one gene demonstrated hypervariable expression per Hansen et al. [11].

**Table 1:** Differentially methylated regions obtained when comparing LCLs with CD40L-activated B cells

List	Number of loci/SNPs	1-kb tiles with data	DMRs	DMRs as % of tiles with data
LCLeQTL	35	34	6	17.65
MSGWAS	201	199	34	17.09
GWAS catalog	51,899	51,151	20,191	39.47
Genome wide	2,881,045	2,648,736	1,216,588	45.93

To determine the source of this differential methylation, we compared methylation values between LCLeQTL, MSGWAS, and GWAS catalog lists for LCLs only or CD40L-activated B cells only. We found only CD40L-activated B cell methylation to be significantly different when comparing immune/B cell loci with the GWAS catalog list (Table 2 and Fig. 1b), suggesting that the lack of DMRs is due to the lower methylation state of LCLeQTL and MSGWAS loci in CD40L-activated B cells rather than LCLs.

**Table 2:** Summary statistics of 1-kb tile methylation

	*LCLeQTL*		*MSGWAS*		*GWAS Catalog*	
	Activated	LCL	Activated	LCL	Activated	LCL
**Maximum**	1.00	1.00	1.00	1.00	1.00	1.00
**Minimum**	0.02	0.02	0.01	0.01	0.00	0.00
**Median**	0.80	0.68	0.85	0.74	0.88	0.68
**First quartile**	0.42	0.27	0.61	0.40	0.79	0.52
**Third quartile**	0.93	0.89	0.93	0.86	0.93	0.83
**IQR**	0.51	0.62	0.31	0.46	0.14	0.31

To confirm that immune loci were specifically underrepresented in EBV demethylation, we sought to determine the number of DMRs using 1-kb tiles centred on loci most highly correlated with whole blood gene expression (nominal *p* value < 10^−20^) derived from the Genotype Tissue Expression (GTEx) Project [23]. We found 15.17% of the 73,771 tiles with data to be differentially methylated. We repeated the analysis with 1-kb tiles centred upon loci most highly correlated with LCL gene expression (nominal *p* value < 10^−20^) from the GTEx project. In this instance, 9477 tiles contained data, with 15.02% of these demonstrating differential methylation.

We next sought to determine whether genes associated with DMRs across the genome were associated with biological gene ontology processes. Using a statistical overrepresentation test with default settings [24] (annotation version 14.1, released March 12, 2019), we found genes proximal to DMRs demonstrated an underrepresentation of B cell and immune activation ontologies (Table 3), consistent with the underrepresentation of DMRs amongst immune and B cell loci (see Supplementary Information 3). The only biological process overrepresented relative to background was multicellular organismal process (GO:0032501), which was not specific to immune or B cell processes.

**Table 3:** Top ten biological processes associated with genes proximal to genome-wide DMRs (for full listing see Supplementary Information 3)

Biological process	Fold enrichment	Raw *P* value	FDR
Positive regulation of lymphocyte activation (GO:0051251)	0.06	7.40E−17	1.33E−13
B cell receptor signaling pathway (GO:0050853)	0.13	1.70E−14	1.53E−11
Phagocytosis (GO:0006909)	0.29	5.61E−10	3.36E−07
Regulation of lymphocyte activation (GO:0051249)	0.29	8.67E−10	3.89E−07
Regulation of leukocyte activation (GO:0002694)	0.31	2.71E−09	9.72E−07
Antigen receptor-mediated signaling pathway (GO:0050851)	0.34	6.22E−09	1.86E−06
Regulation of cell activation (GO:0050865)	0.32	7.86E−09	2.02E−06
Immune response (GO:0006955)	0.58	1.71E−08	3.84E−06
B cell activation (GO:0042113)	0.35	3.01E−08	6.00E−06
Defense response to bacterium (GO:0042742)	0.38	8.60E−08	1.54E−05

### The underrepresentation of DMRs also occurs at MHC regions

HLA haplotypes have previously been shown to have the largest genetic contribution to MS risk. In addition, differential methylation at the MHC region in CD4+ T cells has been found to be associated with MS [25, 26]. We therefore interrogated the MHC region in LCLs and CD40L-activated B cells by examining methylation in 1 kb windows centred upon SNPs in LD with MS HLA risk alleles [27]. We found DMRs to be underrepresented, with 3/17 (17.65%) windows hypomethylated (see Supplementary Information 4).

### The underrepresentation of DMRs is not due specifically to B cell activation

**Fig. 2:**
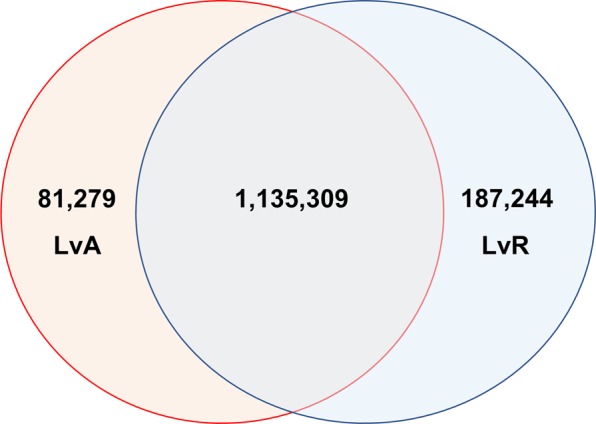
Overlap of genome-wide DMRs for LvA and LvR comparisons based on 1-kb tiles

To determine whether the underrepresentation of immune and B cell loci amongst DMRs was due to the activation state of both LCLs and CD40L-activated B cells, we compared methylation between LCLs and resting B cells from the same individuals. Once again, there was a statistically significant underrepresentation of DMRs amongst LCLeQTL (*p* < 0.01; minimum from random sampling of GWAS Catalog—8 DMRs) and MSGWAS loci (*p* < 0.01; minimum from random sampling of GWAS Catalog—103 DMRs) (Table 4). To determine whether the DMRs occurred at similar regions in both LCL vs CD40L-activated B cells (LvA) and LCL vs resting B cells (LvR), we determined the proportion of overlapping DMRs between the comparisons on a genome-wide basis. Almost all LvA DMRs (93.31%) overlapped with LvR DMRs, whereas 85.84% of LvR DMRs overlapped with LvA DMRs (Fig. 2). We also performed a statistical overrepresentation test on LvR DMRs across genome wide 1-kb tiles. There was a large overlap between GO terms belonging to both LvR and LvA lists (see Supplementary Information 5). Overall, this suggests that the underrepresentation of DMRs amongst immune and B cell loci between LCLs and CD40L-activated B cells is not due primarily to the activation status of these subsets.

**Table 4:** Differentially methylated regions obtained when comparing LCLs with resting B cells

List	Number of loci/SNPs	1-kb tiles with data	DMRs	DMRs as % of tiles with data
LCLeQTL	35	34	7	20.59
MSGWAS	201	199	44	22.11
GWAS catalog	51,899	51,144	22,250	43.50
Genome wide	2,881,045	2,648,736	1,321,140	49.93

### The underrepresentation of DMRs is not due to differential distribution of B cell/immune cell loci in specific gene annotations

**Fig. 3:**
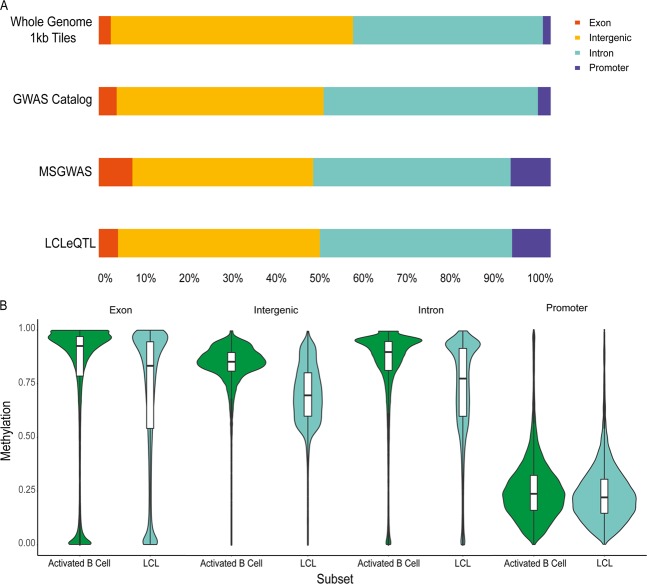
**a** Breakdown of loci overlapping with gene annotations and **b** violin plots of DNA methylation by gene annotation and cell subset

The underrepresentation of DMRs may be due to the differential distribution of these regions amongst various genomic annotations. For example, if specific gene annotations are associated with lower or higher methylation states, this may bias the likelihood of DMRs if loci predominantly overlap with these annotations. Thus, we sought to determine the overlap of loci in each list with genomic annotations, comprising promoters, exons and introns and intergenic regions not otherwise covered by other annotations (Fig. 3). There was no difference in distribution of loci between LCLeQTL and GWAS Catalog lists by Fisher’s exact test (*p* = 0.14), however the distribution was significantly different between MSGWAS and GWAS Catalog lists (*p* = 5.90 × 10^−8^, *χ*^2^ = 36.49, df = 3). Finally, a comparison of the midpoint of whole-genome 1-kb tiles and the GWAS Catalog list showed a significant difference between observed and expected numbers of loci corresponding to each annotation (*p* < 2.20 × 10^−16^, *χ*^2^ = 1651.20, df = 3).

Given the differences in expected proportions between MSGWAS, whole-genome 1-kb tiles and GWAS Catalog loci, we examined the effect of EBV infection on DNA methylation for particular gene annotations. Interestingly, promoter methylation was minimally affected by EBV infection, whereas other gene annotations demonstrated marked hypomethylation due to EBV. Despite this, the relatively small proportion of loci overlapping with promoter regions would not account for the paucity of DMRs in immune and B cell loci. The difference between the proportion of promoters amongst LCLeQTL/MSGWAS loci versus GWAS Catalog loci was ~6%. Even assuming that these 6% were DMRs, the probability of obtaining 7 and 46 DMRs for LCLeQTL and MSGWAS loci would be 0.01 and <0.01, respectively.

### Promoter regions are not hypomethylated in LCLs

Methylation of promoter regions is thought to be closely related to gene expression. Interestingly, despite large areas of hypomethylation in LCLs, promoter regions only appeared to be minimally affected by EBV transformation (see Fig. 3b). Of 13,983 promoters with data, only 99 were differentially methylated (0.71%). A statistical overrepresentation test [24] did not show any statistically significant gene ontology associations.

### Gene expression correlates weakly with differential methylation in an annotation specific manner

**Fig. 4:**
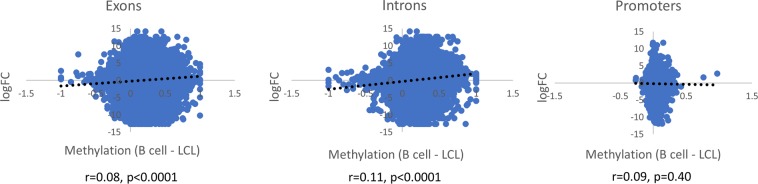
Scatter plots comparing gene expression as a function of DNA methylation differences between LCLs and resting B cells

Having found that the majority of differentially methylated tiles overlap between LvR and LvA conditions, we compared the expression of resting B cells and LCLs from a separate RNA-seq dataset, to determine the extent to which differential expression occurs and to what extent it is correlated with differential methylation. This was done by comparing log transformed relative expression as a function of methylation difference between resting B cells and LCLs by gene annotation i.e., exon, intron, and promoter regions (Fig. 4). Of 14,740 genes with expression level data, 8515 (57.77%) demonstrated at least single log-fold changes in expression level (LCL > resting B cells—2731, LCL < resting B cells 5784). In comparing expression and differential methylation, for exons and introns, points tended to cluster at *x* > 0, consistent with the effect of EBV infection on DNA methylation. For promoters, points clustered around *x* = 0, as expected due to the relatively small effect of EBV on DNA methylation at promoters. The correlation between methylation difference and expression was weakly positive for exons and introns and was not present at all for promoters. We also determined the number of DMRs corresponding to high (>single log-fold) or low or absent (≤single log-fold) expression differences between subsets (Table 5). Regions with high levels of differential expression were more likely to be differentially methylated at the 0.2 level than those with low or no expression differences between subsets (*χ*^2^ test; exons *p* < 0.0001; introns *p* < 0.0001; promoters *p* = 0.0391).

**Table 5:** Rates of differential expression by expression level and annotation

	Exons	Introns	Promoters
≤One log-fold difference in expression	61,482	64,846	4900
% Differentially methylated	7.16%	6.74%	0.67%
>One log-fold difference in expression	78,155	82,312	5318
% Differentially methylated	14.51%	17.80%	1.05%

## Discussion

This study interrogated whole-genome bisulfite sequencing methylomes to determine whether EBV mediated dysregulation of DNA methylation occurs at MS risk loci. Consistent with previous studies, we found widespread dysregulation across the entire genome, with large areas of hypomethylation in LCLs relative to other subsets. Given the persistence and rapid proliferation of LCLs, we expected B cell/immune activation loci to demonstrate differential methylation to a greater extent than other regions. Surprisingly, we found the opposite to be true, that hypomethylation in LCLs at B cell/immune loci occur much less frequently than other regions of the methylome. Further analysis showed that this was due in part to constitutive B cell utilization of these regions, with relative hypomethylation in CD40L-activated B cells compared with other regions. This effect was independent of B cell activation state and distribution of loci amongst gene annotations.

Given that EBV hypomethylation affects 2.18 Gb and including one-third of genes, we would expect by chance that >65 MS risk genes would be in the hypomethylated regions, but only 35 were. Also, of the 47 MS risk SNPs we had earlier identified as associated with gene expression in LCLs [5], only 6 were in a hypomethylated region. Many of these were not coding genes, and the others did not have obvious functions related to EBV infection, compared with the other risk genes (Supplementary Information 6). In addition, a minority of SNPs associated with MHC related MS risk alleles were in hypomethylated regions. This may indicate that the hypomethylation trait is more related to the generation of tumors and immortalization than to the effect of EBV on autoimmune risk. Hansen et al. [11] had established that hypomethylated blocks, and the genes contained within them, overwhelmingly correspond to those seen in cancer, with an overlap of 1.72 GB. In a study of over 2000 tissues, most from tumors, Perez et al. [28] concluded that hypomethylation was shared across tumor types, independent of tumor tissue origin, and distinct from the hypomethylation associated with aging. Although many of the genes required for methylation are encoded in heterochromatic regions which are hypomethylated in EBV, suggesting their reduced expression may reduce effective methylation on proliferation, the hypomethylation in tumor, and LCLs has little overlap with proliferation [11, 12]. The shared hypomethylation between tumors and LCLs appears to be related to chromatin modification, especially due the inhibiting histone modification H3K9me3 [28]. Hansen et al. [11] have suggested the hypomethylation might favor immortalization by generating hypervariable gene expression, enabling selection of cell lines avoiding immune detection and those with apoptosis avoiding transcriptional programs.

A limitation of this study is that we cannot know which MS risk loci, if any, are risk loci because they affect host response to EBV. We also cannot be sure if hypomethylation is different in LCLs from people with MS, especially given that different methylation has been detected in some regions of the uninfected immune cell genome in MS compared with controls [25, 26, 29]. A larger sample size of individuals may reveal more diversity in DMRs. Further, the interaction between methylation and transcription is complex, and risk SNPs may interact with the methylation machinery independently of co-location with DMRs.

Although our data does not indicate a link between MS risk genes and EBV hypomethylation, it is notable that others have suggested that the EBV hypomethylation is associated with immortalization (see above). EBV immortalization of forbidden clones targeting myelin, especially through lymph node processes, has been considered a likely mechanism driving the association of EBV with MS [30]. Genetic and environmental factors controlling this immortalization may drive difference in risk between individuals. The genetic component could be due to germline variation or somatic variation. Future work targeting variation between individuals and the process of EBV hypomethylation may identify genetic variation important in the contribution of EBV infection to MS pathogenesis.

## Supplementary information

List of Supplementary Information

Supplementary Information 1

Supplementary Information 2

Supplementary Information 3

Supplementary Information 4

Supplementary Information 5

Supplementary Information 6
