# Supplementary material for: Transcribed B lymphocyte genes and multiple sclerosis risk genes are underrepresented in Epstein–Barr Virus hypomethylated regions
Source: Genes Immun. 2019 Oct 16;21(2):91–9. doi: 10.1038/s41435-019-0089-5 (PMC7182534; doi:10.1038/s41435-019-0089-5)
Supplement: Supplementary file 1 — List of Supplementary Information [file 41435_2019_89_MOESM1_ESM.docx]

**Supplementary Information**

Supplementary Information 1. Bisulfite conversion rates, coverage and alignment statistics by subject and cell subset (.xlsx format)

Supplementary Information 2. Coverage statistics for merged methcounts data by subset (.xlsx format)

Supplementary Information 3. GO process terms associated with differentially methylated 1kb tiles for CD40L activated B cells and LCLs (.xlsx format)

Supplementary Information 4. SNPs in LD with MS MHC risk alleles and differential methylation status in LCLs vs CD40L activated B cells (.xlsx format)

Supplementary Information 5. GO process terms associated with differentially methylated 1kb tiles for resting B cells and LCLs (.xlsx format)

Supplementary Information 6. Genes associated with differentially methylated loci in MSGWAS and LCLeQTL lists (.xlsx format)
